# Communication practices that encourage and constrain shared decision making in health‐care encounters: Systematic review of conversation analytic research

**DOI:** 10.1111/hex.12557

**Published:** 2017-05-18

**Authors:** Victoria Land, Ruth Parry, Jane Seymour

**Affiliations:** ^1^ University of Nottingham Nottingham UK; ^2^ University of Sheffield Sheffield UK

**Keywords:** conversation analysis, medical interaction, patient choice, patient participation, shared decision making, systematic review

## Abstract

**Background:**

Shared decision making (SDM) is generally treated as good practice in health‐care interactions. Conversation analytic research has yielded detailed findings about decision making in health‐care encounters.

**Objective:**

To map decision making communication practices relevant to health‐care outcomes in face‐to‐face interactions yielded by prior conversation analyses, and to examine their function in relation to SDM.

**Search strategy:**

We searched nine electronic databases (last search November 2016) and our own and other academics' collections.

**Inclusion criteria:**

Published conversation analyses (no restriction on publication dates) using recordings of health‐care encounters in English where the patient (and/or companion) was present and where the data and analysis focused on health/illness‐related decision making.

**Data extraction and synthesis:**

We extracted study characteristics, aims, findings relating to communication practices, how these functioned in relation to SDM, and internal/external validity issues. We synthesised findings aggregatively.

**Results:**

Twenty‐eight publications met the inclusion criteria. We sorted findings into 13 types of communication practices and organized these in relation to four elements of decision‐making sequences: (i) broaching decision making; (ii) putting forward a course of action; (iii) committing or not (to the action put forward); and (iv) HCPs' responses to patients' resistance or withholding of commitment. Patients have limited opportunities to influence decision making. HCPs' practices may constrain or encourage this participation.

**Conclusions:**

Patients, companions and HCPs together treat and undertake decision making as shared, though to varying degrees. Even for non‐negotiable treatment trajectories, the spirit of SDM can be invoked through practices that encourage participation (eg by bringing the patient towards shared understanding of the decision's rationale).

## 1 BACKGROUND

Shared decision making (SDM) ‘…is a process in which clinicians and patients work together…with the aim of reaching mutual agreement on the best course of action’ (p.2).^1^ SDM is advocated as an ideal model of health‐care decision making^2^, ^3^ and is associated with better health‐care efficiency, quality and outcomes and highly valued by patients.^4^, ^5^, ^6^ However, implementation is not universal despite HCPs' claims to be doing SDM.^7^, ^8^

SDM involves engaging in decision making or plan‐making collaboratively wherein both patient (and/or companion) and HCP contribute. We drew on the conceptual framework proposed by Entwistle and Watt^9^ which extends beyond a focus on the “selection from a menu of health‐care options” (p.276) and, therefore, is more broadly applicable to all decisions (ie spanning those with multiple reasonable courses of action *and* where there is only one course). It includes, but is not restricted to, recognition of patients' perspectives and contributions, being committed to a goal/activity, communicating significant issues and being informed.

Arguably, the concept of SDM has received more attention than its actual implementation in real‐life health‐care episodes. To help cast some light, we synthesised one body of evidence—that from conversation analytic studies of health‐care encounters. Conversation analysis (CA) is a systematic and methodologically distinctive approach to studying interaction. It elucidates both the structural forms and the functional consequences of communication practices by studying recordings of actual interactions.^10^, ^11^ The recording process affects the interaction to some extent,^12^ but considerable evidence suggests this does not preclude valid, useful findings.^13^ CA does not try to understand communication by imputing psychological states; rather, it builds understandings of what people accomplish (together) through communication.

It is reasonable to understand *all* communications during health‐care encounters as integral to decision making. However, in this review, we purposely narrow the focus to commitment points: where it becomes relevant for patients to commit—or not—to a course of action (eg immediately after a HCP's proposal or suggestion). This is because decisions are internal matters that can only be gotten at through verbal claims and observable behaviours (ie commitment). We examine communication practices that happen during, shortly before and shortly after commitment points. CA research on communication in relation to health‐care decision making is not comprehensive—some settings and decision types have been extensively studied, others minimally or not at all.

Communications included a variety of health‐care matters: prescribing/altering pharmaceuticals, surgery, vaccination, psychotherapeutic or radiological intervention(s) or equipment; ordering/offering clinical/screening tests; setting therapeutic goal(s); and lifestyle adjustments. Our key objectives were as follows:

1. To identify communication practices entailed in decision making in health‐care interactions.
2. To highlight patients'/companions' actions which contribute to their participation in decision making. Participation includes patients/companions having opportunity to discuss and/or influence decision making, having their points of view taken into consideration and/or opportunities for consultation and/or negotiation.
3. To examine how HCPs' practices encourage and constrain participation.

## 2 METHODS

We used an approach developed previously for systematically reviewing conversation analytic and discourse analytic research.^14^ The rationale and process of this reviewing approach are described in a dedicated paper.^15^ We used an aggregate approach to map findings across the structure that emerged (rather than undertaking a re‐analysis).

### 2.1 Study selection

One author (VL) undertook searching and initial screening of titles and abstracts and excluded publications clearly not meeting these criteria:

1. Audio/audio‐visual recording of naturalistic health‐care interactions with co‐present patients/companions.
2. In English.
3. Both data and analysis examined broaching, considering, planning and/or deciding health/illness‐related actions.
4. CA as a primary analytic approach.
5. Published in books or peer‐reviewed journals (no date restrictions).

**Figure 1:**
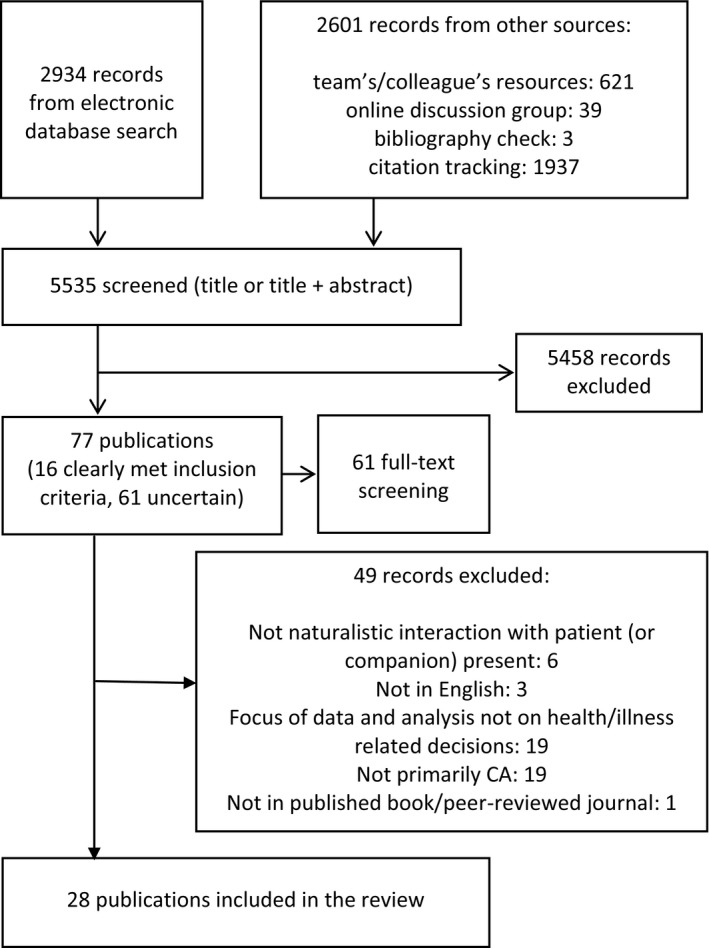
Flowchart depicting searching, screening and inclusion of studies

Remaining records (see Figure 1) were independently assessed by two reviewers (VL and RP); disagreements were resolved through discussion.

### 2.2 Information sources

Nine electronic databases were searched (last search November 2016): Amed; ASSIA; CINAHL; Embase; ISI Web of Science; Medline; PsycINFO; Scopus; and Sociological Abstracts CSA (Table 1 details search terms). Following contemporary guidance,^16^, ^17^ we searched additional sources: our own and other academics' reference collections; specialist bibliographies; and online discussion groups.

**Table 1:** Search terms for database search

Database	Word group 1	Word group 2	Word group 3	Word group 4	Word group 5
Amed; ASSIA; CINAHL; Embase; ISI Web of Science; Medline; Sociological Abstracts CSA	communicat* OR interact*	decision* OR negotiat* OR choice	discourse OR conversation	clinical OR medical OR healthcare OR doctor	AND NOT biolog* OR neuro* OR gene*
PsycINFO; Scopus	communicat* OR interact*	decision* OR negotiat* OR choice	discourse‐analysis OR conversation‐analysis	clinical OR medical OR healthcare OR doctor	AND NOT biolog* OR neuro* OR gene*

### 2.3 Data collection, appraisal and synthesis

We developed, piloted and then used a customized data extraction form^15^ to extract study characteristics, aims, findings relating to communication practices, how these functioned in relation to SDM and internal/external validity issues for study appraisal. We synthesized findings aggregately through discussion within the research team and via consultations with clinicians and researchers (both individually with academics/colleagues and also at seminars for sharing our work‐in‐progress).

## 3 FINDINGS

Twenty‐eight records^18^, ^19^, ^20^, ^21^, ^22^, ^23^, ^24^, ^25^, ^26^, ^27^, ^28^, ^29^, ^30^, ^31^, ^32^, ^33^, ^34^, ^35^, ^36^, ^37^, ^38^, ^39^, ^40^, ^41^, ^42^, ^43^, ^44^, ^45^ were identified (see Table 2 for publication characteristics). We organized findings in chronological order: actions prior to commitment point(s) being reached (termed “broaching”); putting forward a course of action (commitment becomes relevant); how patients convey commitment (or not); and HCPs' responses to patients' resistance or withholding of commitment. Table 3 summarizes the practices, their functions, and the settings and publications in which they were documented.

**Table 2:** Characteristics of 28 included publications and their contributions to the findings of the review

Study, Country	Academic field of publication	Data characteristics: Setting, participants Size of data set Whether data were audio only or audio visual	Contributes to review findings in terms of:
Angell & Bolden (2015) USA	Sociology	36 clients in an assertive community treatment (ACT) programme for people with serious and prolonged psychiatric disorders (eg schizophrenia, bipolar disorder) with a team psychiatrist 36 interactions Audio only	*Subtle* lobbying by patient (or companion) for a treatment/test *before* a commitment point has been reached Includes instances of practitioners putting forward an affirmative single course of action Practitioner rules out a single option which may be considered potential primary treatment Orientation to patient agreement as necessary for progressing to the next phase (and withholding that agreement is a means of passively resisting the course of action put forward) Practitioner purses commitment after patient resistance without changing course of action put forward
Barnard et al. (2010) UK	Clinical/medical	18‐bed neurological rehabilitation unit in a large metropolitan hospital in London, six patients (three with multiple sclerosis, two with spinal cord lesions, one stroke patient), four physiotherapists, four occupational therapists, four nurses, one speech and language therapist and 1 neuropsychologist. Six interactions Audio visual	Practitioners eliciting patients’ perspectives prior to putting forward a course of action Includes instances of practitioners putting forward an affirmative single course of action Orientation to patient agreement as necessary for progressing to the next phase Analytic focus on withholding agreement as a form of passive resistance Active resistance through questions/concerns pertaining to the medical problem and/or proposed treatment
Clark & Hudak, (2011) Canada	Language/linguistic	Two metropolitan hospitals in a major Canadian city 14 orthopaedic surgeons and 121 patients Audio only	Encouraging patient agreement prior to putting forward a course of action Includes instances of practitioners putting forward an affirmative single course of action Practitioner rules out a single option which may be considered potential primary treatment Orientation to patient agreement as necessary for progressing to the next phase (and withholding that agreement is a means of passively resisting the course of action put forward) Practitioner pursues commitment after patient resistance without changing course of action put forward
Collins (2005) UK	Clinical/medical	GP surgeries, 23 patients with diabetes, six doctors and five nurses Total 38 consultations Audio visual	Includes instances of practitioners putting forward an affirmative single course of action
Collins et al. (2005) UK	Sociology	Five areas of clinical practice—family planning, homoeopathy, clinical cancer genetics, ENT oncology, general practice management of diabetes (although data for this paper came from the last two settings only). 114 patients and 47 health professionals From the total data set of 168 consultations, 80 were identified as having decision‐making sequences (45 of which analysed in detail for this study) Audio visual where consent was given	Flagging up the approaching decision point Practitioners eliciting patients’ perspectives prior to putting forward a course of action Includes instances of practitioners putting forward an affirmative single course of action Practitioner puts forward multiple options
Costello & Roberts (2001) USA	Interdisciplinary health and social sciences	Two university based oncology clinics, 14 physicians and 37 patients 37 consultations Audio only for 21 interactions and audio visual for 16 interactions	Includes instances of practitioners putting forward an affirmative single course of action Explicit analytic focus on patients’ commitments to courses of action as necessary for progressing to the next stage Analytic focus on withholding agreement as a form of passive resistance Active resistance by explicitly advocating for a specific treatment after a commitment point has been reached Practitioner modifies course of action put forward after patient resistance Decision is left open by deferral or opportunity to review in the future
Gafaranga & Britten (2007) UK	Interdisciplinary health and social sciences	Consultations from general practice Total data set not stated, number of episodes analysed not stated Audio only	Explicit analytic focus on patients’ commitments to courses of action as necessary for progressing to the next stage
Gill (2005) USA	Language/linguistic	Hospital‐based outpatient clinic Single‐case analysis taken from a data set of 15 interactions Audio visual	*Subtle* lobbying by patient (or companion) for a treatment/test *before* a commitment point has been reached Includes instances of practitioners putting forward an affirmative single course of action
Gill et al. (2001) USA	Language/linguistic	Hospital‐based outpatient clinic Single‐case analysis Audio visual	*Subtle* lobbying by patient (or companion) for a treatment/test *before* a commitment point has been reached Includes instances of practitioners putting forward an affirmative single course of action
Hudak et al. (2011) Canada	Sociology	Two academic hospitals in a major Canadian city 14 orthopaedic surgeons and 121 patients Audio only	Encouraging patient agreement prior to putting forward a course of action Includes instances of practitioners putting forward an affirmative single course of action Practitioner rules out a single option which may be considered potential primary treatment Orientation to patient agreement as necessary for progressing to the next phase (and withholding that agreement is a means of passively resisting the course of action put forward)
Hudak et al. (2012) Canada	Interdisciplinary health and social sciences	Two academic hospitals in a major Canadian city 14 orthopaedic surgeons and 121 patients Audio only	Encouraging patient agreement prior to putting forward a course of action Includes instances of practitioners putting forward an affirmative single course of action Practitioner rules out a single option which may be considered potential primary treatment Orientation to patient agreement as necessary for progressing to the next phase (and withholding that agreement is a means of passively resisting the course of action put forward)
Koenig (2011) USA	Sociology	Acute visits to 10 clinics in Western USA. Participants were internists and adult patients 100 consultations Audio visual	Includes instances of practitioners putting forward an affirmative single course of action Practitioner rules out a single option which may be considered potential primary treatment Explicit analytic focus on patients’ commitments to courses of action as necessary for progressing to the next stage Analytic focus on withholding agreement as a form of passive resistance Active resistance through questions/concerns pertaining to the medical problem and/or proposed treatment Practitioner pursues commitment after patient resistance without changing course of action put forward Practitioner modifies course of action put forward after patient resistance
Miller et al. (1992) USA	Clinical/medical	Medical intensive care unit of a tertiary care centre. Family members included a combination of spouses (five cases), adult children (eight cases) and siblings (seven cases). Patients themselves participated in seven instances. Seven attending physicians, five fellows and four residents were involved in one or more meetings Data set comprises 15 meetings Audio only	Practitioners eliciting patients’ perspectives prior to putting forward a course of action Includes instances of practitioners putting forward an affirmative single course of action Practitioner puts forward multiple options (subsidiary point) Explicit analytic focus on patients’ commitments to courses of action as necessary for progressing to the next stage Orientation to withholding agreement as a means of passively resisting the course of action put forward Decision is left open by deferral or opportunity to review in the future
Opel et al. (2012) USA	Clinical/medical	Health supervision visits in which vaccination is discussed from five paediatric practices. Seven practitioners (paediatricians but may include one paediatric nurse) and 20 vaccine‐hesitant parents. 20 consultations Audio visual	Practitioners eliciting patients’ perspectives prior to putting forward a course of action Includes instances of practitioners putting forward an affirmative single course of action Explicit analytic focus on patients’ commitments to courses of action as necessary for progressing to the next stage Analytic focus on withholding agreement as a form of passive resistance Active resistance through questions/concerns pertaining to the medical problem and/or proposed treatment Active resistance by explicitly advocating for a specific treatment after a commitment point has been reached Practitioner pursues commitment after patient resistance without changing course of action put forward Decision is left open by deferral or opportunity to review in the future
Parry (2004) UK	Clinical/medical	Physiotherapy “gyms” in four UK hospitals. 21 Patients and 10 physiotherapists. 74 physiotherapy sessions, eight of these have involve goal setting Audio visual	Practitioners eliciting patients’ perspectives prior to putting forward a course of action Includes instances of practitioners putting forward an affirmative single course of action Orientation to patient agreement as necessary for progressing to the next phase (and withholding that agreement is a means of passively resisting the course of action put forward) Practitioner pursues commitment after patient resistance without changing course of action put forward
Parry (2009) UK	Sociology	Neurological physiotherapy sessions in two rehabilitation units. 12 physiotherapists and 21 patients. 41 physiotherapy sessions Audio visual	Encouraging patient agreement prior to putting forward a course of action Includes instances of practitioners putting forward an affirmative single course of action Orientation to patient agreement as necessary for progressing to the next phase (and withholding that agreement is a means of passively resisting the course of action put forward)
Pilnick (2004) UK	Language/linguistic	Community and hospital antenatal clinics. 14 pregnant women (eight from affluent suburban area, six from less affluent inner city area) 14 pre‐screening consultations and 14 post‐consultations Audio only	Includes instances of practitioners putting forward an affirmative single course of action
Pilnick (2008) UK	Sociology	Community and hospital antenatal clinics. 14 pregnant women (eight from affluent suburban area, six from less affluent inner city area) 14 pre‐screening consultations and 14 post‐consultations Audio only	Includes instances of practitioners putting forward an affirmative single course of action
Quirk et al. (2012) UK	Sociology	Two NHS mental health services, nine consultant psychiatrists and 92 patients in outpatient consultations where antipsychotic medications were discussed 92 interactions Audio only	Flagging up the approaching decision point Includes instances of practitioners putting forward an affirmative single course of action Practitioner puts forward multiple options Orientation to patient agreement as necessary for progressing to the next phase (and withholding that agreement is a means of passively resisting the course of action put forward) Active resistance by explicitly advocating for a specific treatment after a commitment point has been reached Practitioner pursues commitment after patient resistance without changing course of action put forward Practitioner modifies course of action put forward after patient resistance
Roberts (1999) USA	Language/linguistic	Oncology units in two hospitals, 23 patients who have undergone surgery for breast cancer and the oncologists they consult with 23 interactions Audio only	Encouraging patient agreement prior to putting forward a course of action Includes instances of practitioners putting forward an affirmative single course of action Explicit analytic focus on patients’ commitments to courses of action as necessary for progressing to the next stage Analytic focus on withholding agreement as a form of passive resistance Active resistance through questions/concerns pertaining to the medical problem and/or proposed treatment Practitioner pursues commitment after patient resistance without changing course of action put forward
Shaw et al. (2016) UK		31 families in discussions with staff in a neonatal intensive care unit This study is based on 16 conversations involving nine families and six consultants Audio only	Practitioners eliciting patients’ perspectives prior to putting forward a course of action Encouraging patient agreement prior to putting forward a course of action Includes instances of practitioners putting forward an affirmative single course of action Practitioner puts forward multiple options Orientation to patient agreement as necessary for progressing to the next phase (and withholding that agreement is a means of passively resisting the course of action put forward) Active resistance through questions/concerns pertaining to the medical problem and/or proposed treatment
Stivers (2005a) USA	Sociology	27 paediatric practices, parents and children seeking medical attention for upper respiratory illness symptoms in consultation with 38 paediatricians The total data set includes 540 interactions. This study is based on a subset of 309 of these interactions. Audio visual	Includes instances of practitioners putting forward an affirmative single course of action Practitioner rules out a single option which may be considered potential primary treatment Explicit analytic focus on patients’ commitments to courses of action as necessary for progressing to the next stage Analytic focus on withholding agreement as a form of passive resistance Active resistance by explicitly advocating for a specific treatment after a commitment point has been reached
Stivers (2005b) USA	Interdisciplinary health and social sciences	Two settings involving acute care paediatric encounters, plus some additional data from possibly a third location. Parents and children seeking medical attention for upper respiratory illness symptoms in consultation with 14 paediatricians 360 interactions (plus some additional interactions recorded at a later date, two of these were used in this study) Audio and audio visual	Includes instances of practitioners putting forward an affirmative single course of action Explicit analytic focus on patients’ commitments to courses of action as necessary for progressing to the next stage Analytic focus on withholding agreement as a form of passive resistance Active resistance through questions/concerns pertaining to the medical problem and/or proposed treatment Practitioner pursues commitment after patient resistance without changing course of action put forward Practitioner modifies course of action put forward after patient resistance Decision is left open by deferral or opportunity to review in the future
Stivers (2002) USA	Sociology	Six private paediatric practices 360 interactions Audio and audio visual	*Subtle* lobbying by patient (or companion) for a treatment/test *before* a commitment point has been reached Includes instances of practitioners putting forward an affirmative single course of action Practitioner rules out a single option which may be considered potential primary treatment Orientation to patient agreement as necessary for progressing to the next phase (and withholding that agreement is a means of passively resisting the course of action put forward) Practitioner modifies course of action put forward after patient resistance
Stivers (2007) USA	Language/linguistic	34 paediatric practices, 54 paediatricians and 882 parent/patients 882 interactions 295 recordings were audio only; 587 were audio visual	*Subtle* lobbying by patient (or companion) for a treatment/test *before* a commitment point has been reached Includes instances of practitioners putting forward an affirmative single course of action Practitioner rules out a single option which may be considered potential primary treatment Explicit analytic focus on patients’ commitments to courses of action as necessary for progressing to the next stage Analytic focus on withholding agreement as a form of passive resistance Active resistance by explicitly advocating for a specific treatment after a commitment point has been reached
Tapsell (1997) Australia	Interdisciplinary health and social sciences	A dietary clinic at a major regional hospital in New South Wales, 19 student dieticians and 30 clients (students’ supervisors were also present) 30 interactions Audio only	Practitioners eliciting patients’ perspectives prior to putting forward a course of action Includes instances of practitioners putting forward an affirmative single course of action Explicit analytic focus on patients’ commitments to courses of action as necessary for progressing to the next stage Analytic focus on withholding agreement as a form of passive resistance
Toerien et al. (2013) UK	Sociology	Two hospital‐based outpatient clinics in the UK, one neurologist and 13 patients 13 interactions Audio only	Flagging up the approaching decision point Includes instances of practitioners putting forward an affirmative single course of action Practitioner puts forward multiple options Explicit analytic focus on patients’ commitments to courses of action as necessary for progressing to the next stage Orientation to withholding agreement as a means of passively resisting the course of action put forward
Toerien et al. (2011) UK	Clinical/medical	Two hospital‐based outpatient clinics in the UK, one neurologist and 13 patients (11 of these patients were accompanied) 13 interactions Audio only	Flagging up the approaching decision point Includes instances of practitioners putting forward an affirmative single course of action Practitioner puts forward multiple options Orientation to patient agreement as necessary for progressing to the next phase (and withholding that agreement is a means of passively resisting the course of action put forward)

Across the 28 publications, the settings included were as follows: eight primary care, three neurorehabilitation, three orthopaedic clinics, two oncology clinics, two outpatient clinics, two antenatal clinics, two mental health, two outpatient epilepsy, two ICUs (one neonatal), one ENT oncology and primary care diabetes, and one dietician clinic.

**Table 3:** Summary of communication practices that encourage and constrain decision making in health‐care encounters

Practice	Phase of consultation	Description of the practice	Function	Number of publications practice is documented	Settings in which practice has been documented
Flagging up	Broaching: actions occurring prior to any commitment point being reached	HCPs flag up an approaching decision point by making an announcement.	These do not stipulate any specific course of action, so they work to encourage patients to move into the activity of deciding, but do not push for one particular decision outcome.	Documented in 4 publications^22^, ^36^, ^44^, ^45^	2 epilepsy; 1 mental health; 1 diabetes/ENT oncology;
Eliciting patient perspectives prior to putting forward a course of action	Broaching: actions occurring prior to any commitment point being reached	A HCP elicits a patient's perspective or preference regarding a possible course of action before the conversation moves to actual decision making.	This practice indicates the nature of the possible upcoming course of action and provides an opportunity to bring to the surface a patient's/companion's views prior to commitment becoming relevant. This may be particularly useful in delicate cases, in cases in which stakes are high or when resistance is likely.	Documented in 7 publications^19^, ^22^, ^30^, ^31^, ^32^, ^38^, ^43^	2 neurorehabilitation; 2 ICU; 1 primary care; 1 diabetes/ENT oncology; 1 dietician
Encouraging patient agreement	Broaching: actions occurring prior to any commitment point being reached	HCPs use practices such as long turns; “brightside” formulations; logical inferences; general case descriptions; and accounting prior to producing a recommendation.	Used particularly when the recommendation is liable to resistance or counter to patients’ expectations, these practices function to achieve patient alignment in a potentially challenging environment.	Documented in 6 publications^20^, ^27^, ^28^, ^33^, ^37^, ^38^	3 orthopaedic; 1 neurorehabilitation; 1 oncology; 1 ICU
Patient lobbying for specific treatment prior to commitment point	Broaching: actions occurring prior to any commitment point being reached	Prior to HCPs referring to a specific course of action or making commitment relevant, patients make reference to a particular course of action.	With this subtle lobbying, patients seek pre‐emptively to influence the treatment trajectory.	Documented in 5 publications^18^, ^25^, ^26^, ^41^, ^42^	2 outpatient clinics; 2 primary care; 1 mental health
Single option	Putting forward the course of action (the commitment point)	A HCP puts forward a single course of action. This may be done with an announcement, a recommendation, a suggestion, an offer, etc. which have varying levels of assumption that the patient should/will follow the course of action.	These turns make relevant a commitment to that course of action or some activity to avoid commitment from the patient. Even when openly phrased, the course of action put forward is likely to be heard as HCP‐endorsed.	Documented in 27 publications^18^, ^19^, ^20^, ^21^, ^22^, ^23^, ^25^, ^26^, ^27^, ^28^, ^29^, ^30^, ^31^, ^32^, ^33^, ^34^, ^35^, ^36^, ^37^, ^38^, ^39^, ^40^, ^41^, ^42^, ^43^, ^44^, ^45^	7 primary care; 3 neurorehabilitation; 3 orthopaedic; 2 oncology; 2 outpatient clinics; 2 antenatal; 2 mental health; 2 epilepsy; 2 ICU (one neonatal); 1 diabetes/ENT oncology; 1 dietician
Ruling out a single option (primary treatment)	Putting forward the course of action (the commitment point)	A HCP may specifically rule out a particular option. This is generally less straightforward—both its design and reception—than affirmatively putting forward a course of action.	By ruling out, the HCP produces the treatment that is ruled out as known to the patient, expectable and also possibly the preferred treatment option.	Documented in 8 publications^18^, ^20^, ^27^, ^28^, ^29^, ^39^, ^41^, ^42^	4 primary care; 3 orthopaedic; 1 mental health
Multiple options	Putting forward the course of action (the commitment point)	HCPs may put forward multiple options from the outset (rather than offering options in response to patients withholding/resisting commitment). The options may be fairly neutral or may display a strong or weak stance towards a particular option.	This practice (ostensibly) provides opportunity for patient participation. However, if the options are “shaded” or omit options, this practice may be a vehicle for recommending rather than offering choice.	Documented as a primary finding in 5 publications^22^, ^36^, ^38^, ^44^, ^45^ and subsidiary finding in 1^30^	Primary: 2 epilepsy; 1 diabetes/ENT oncology; 1 mental health; 1 ICU Subsidiary: 1 ICU
Committing	Committing or not	A single option makes relevant a commitment, and a list makes relevant a selection.	This practice makes relevant patient involvement in reaching a decision as patients/companions and HCPs jointly orient to patient commitment as the necessary next action.	Documented explicitly in 11 publications^23^, ^24^, ^29^, ^30^, ^31^, ^37^, ^39^, ^40^, ^42^, ^43^, ^44^ and in a further 11 publications^18^, ^19^, ^20^, ^27^, ^28^, ^32^, ^33^, ^36^, ^38^, ^41^, ^45^ agreement as the necessary next step is presumed	Explicit: 6 primary care; 2 oncology; 1 epilepsy; 1 ICU; 1 dietician Presumed next step: 3 neurorehabilitation; 3 orthopaedic; 2 mental health; 1 epilepsy; 1 ICU; 1 primary care
Withholding commitment	Committing or not	Patients/companions may withhold commitment through silence or very weak commitment.	This halts progression of the consultation and implies commitment is problematic but does not specify the nature of the problem. This is not indicative of definite or enduring resistance: there may be obstacles to overcome before the patient commits.	Documented explicitly in 9 publications^19^, ^23^, ^29^, ^31^, ^37^, ^39^, ^40^, ^42^, ^43^ and implicitly in a further 12 publications^18^, ^20^, ^27^, ^28^, ^30^, ^32^, ^33^, ^36^, ^38^, ^41^, ^44^, ^45^	Explicit: 5 primary care; 2 oncology; 1 dietician; 1 neurorehabilitation Implicit: 3 orthopaedic; 2 mental health; 2 ICU; 2 neurorehabilitation; 2 epilepsy; 1 primary care
Active resistance: questions/concerns	Committing or not	A patient or their companion may move into active resistance by raising questions or concerns with the option(s) put forward	This is an escalation of resistance from withholding commitment. With these practices, patients indicate the nature of the problem that is an obstacle to commitment and make relevant some response from the HCP to address their concerns.	Documented in 6 publications^19^, ^29^, ^31^, ^37^, ^38^, ^40^	3 primary care; 1 oncology; 1 neurorehabilitation; 1 ICU
Active resistance: advocating for some alternative after reaching a commitment point	Committing or not	A patient or their companion may actively resist by advocating for an alternative course of action after the HCP has already put forward a course of action.	This is the stronger form of active resistance. Patients and their companions treat themselves as active in determining the decision with this practice.	Documented in 5 publications^23^, ^31^, ^36^, ^39^, ^42^	3 primary care; 1 oncology; 1 mental health
Pursue agreement without changing course	Responding to the patient's response to the list, option or rule out	HCPs pursue commitment after resistance but without altering the option(s) put forward. This may take the form of responding to the obstacles put forward by the patient/companion or HCPs may pursue commitment without engaging in this issues raised by the patient/companion.	Where HCPs engage with the barriers to commitment, patient participation is evident even though the treatment trajectory has not changed.	Documented in 8 publications^18^, ^20^, ^29^, ^31^, ^32^, ^36^, ^37^, ^40^	3 primary care; 2 mental health; 1 orthopaedic; 1 neurorehabilitation; 1 oncology
Modify the potential course of action (pursuing agreement by changing course)	Responding to the patient's response to the list, option or rule out	HCPs may attend to patients’/companions’ resistance by modifying the recommendation. This modification may involve declining treatment or taking a lower dose or agreeing to an alternative treatment.	These modifications show patients’/companions’ agency through having a direct influence on the treatment trajectory.	Documented in 5 publications^23^, ^29^, ^36^, ^40^, ^41^	3 primary care; 1 oncology; 1 mental health
Leave the decision open	Responding to the patient's response to the list, option or rule out	HCPs may deal with patients’/companions’ resistance by leaving the decision open, either by deferring it until another time or by offering to review and revise it at a later date.	HCPs may use these options to show they have taken the patient's/companion's concerns seriously and are open to changing the options on offer in the future.	Documented in 4 publications^23^, ^30^, ^31^, ^40^	2 primary care; 1 oncology; 1 ICU

### 3.1 Broaching decision making: actions occurring prior to any commitment point being reached

We term activities relevant to decision making but before a commitment point is reached broaching activities. Four ways of broaching were documented: flagging up that a commitment point is approaching; eliciting patient perspectives about decisions; encouraging patient agreement with proposals; and patient lobbying for a specific treatment/test.

#### 3.1.1 Flagging up

**Figure 2:**
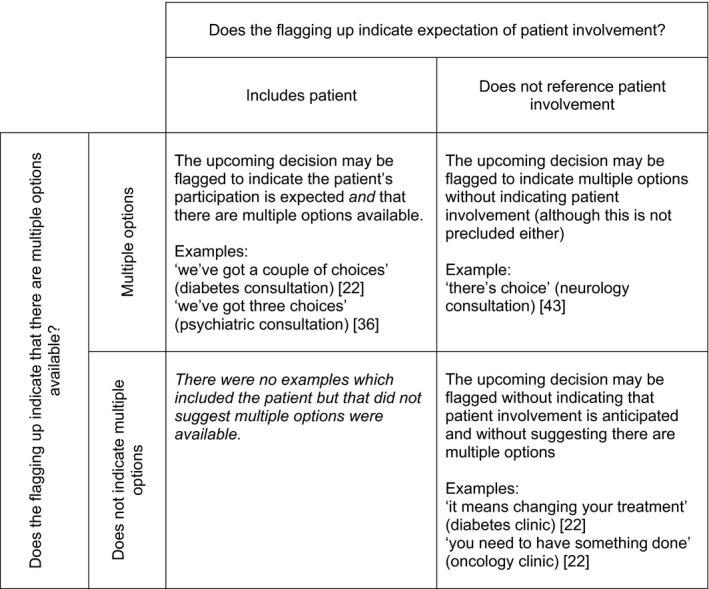
Flagging up an upcoming decision may indicate multiple options and/or the expectation of patient involvement

In four publications,^22^, ^36^, ^44^, ^45^ HCPs make an announcement to indicate an approaching commitment point. This “flagging up” does not stipulate any course of action; it encourages patients to move into the activity of deciding but does not push for one specific outcome. Nevertheless, announcements can indicate other aspects, including whether there are multiple options or whose decision it is (Figure 2).

#### 3.1.2 Eliciting patient perspectives prior to putting forward a course of action

Seven publications^19^, ^22^, ^30^, ^31^, ^32^, ^38^, ^43^ documented HCPs eliciting patients'/companions' perspectives/preferences regarding possible courses of action before actual decision making. In an ICU study, physicians sought patients' views and relatives' understandings of patients' wishes concerning the continuation or withdrawal of life‐sustaining treatment:^30^


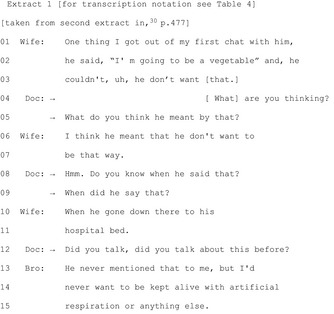


**Table 4:** Transcription key

.	Falling intonation
,	Continuing intonation
?	Rising intonation
¿	Slightly rising intonation
↑	Rise in pitch
↓	Fall in pitch
.hh	Audible inbreath
underlining	Produced with emphasis
[]	Overlapping talk
=	Contiguous talk
(0.5)	Silence—the number represents the length of silence in seconds
(.)	Silence less than a tenth of a second
:	Stretch on preceding sound
◦ ◦	Talk within symbols is quieter than surrounding talk
CAPITALS	Talk louder than surrounding talk
< >	Talk within symbols is slower than surrounding talk
> <	Talk within symbols is faster than surrounding talk
‐	Preceding sound is cut off
(())	Non‐lexical occurrences

Extract 1 shows multiple elicitations (lines 4‐5, 8‐9, 12) as the physician seeks the family's perspectives.

A study of dietetic students provides more evidence on eliciting patient perspectives before advancing a course of action.^43^ When students elicited clients' perspectives about dietary changes before suggesting changes and *followed up their clients' answers with further questions,* clients were more likely to commit to those decisions.^43^ Soliciting patients' views can contribute to a “bilateral” approach^22^—one that seeks to incorporate the patient in decision making by consistently seeking the patient's perspective and building “the next phase of the decision making on the patient's answers” (p.2614).

Eliciting patients' (or companions') views—of their problems and desires for treatment—are a key aspect of SDM**.** However, elicitation is not sufficient to indicate participation. In relation to physiotherapy goals, a study found “[e]liciting and incorporating patients' views and setting goals are demanding and potentially time‐consuming activities” (p.679), and even when physiotherapists sought patients' viewpoints, they may be neither forthcoming nor relevant/useful in formulating appropriate goals.^32^

In sum, practices for eliciting patients'/companions' perspectives regarding a potential course of action can occur prior to a point where commitment to that action is relevant. They indicate the nature of the upcoming course of action and provide opportunities to bring to the surface patients'/companions' views prior to commitment becoming relevant. This might be particularly useful in delicate cases, when stakes are high or when resistance is likely. Finally, institutional pressures may result in difficulties in incorporating patient views into subsequent actions.

#### 3.1.3 Encouraging patient agreement

There are several practices used in isolation or combination in service of achieving patient agreement with a not‐yet‐specified course of action while indicating the nature of that action. Six publications^20^, ^27^, ^28^, ^33^, ^37^, ^38^ document these practices (although three from one programme of research) from various settings: orthopaedics; oncology; neonatal ICU; and neurological physiotherapy. These practices include long turns, “brightside” formulations, logical inferences, general case descriptions and accounting.

##### Long turns

Orthopaedic surgeons projected longer turns through prefacing or requiring an element of the initial talk to be unpacked or by inserting parenthetical talk, thereby minimizing a patient's opportunity to disagree with the parenthetical information.^20^ These long turns, found in non‐surgical recommendations, allow surgeons to “concurrently manage multiple (competing) contingencies and actively work to anticipate and pre‐empt possible problems with the action underway” (p.397).

##### “Brightside” formulations

Surgeons used “brightside” formulations—by making his/her own positive evaluation (Extract 2) or, more powerfully, by drawing on a patient‐reported positive (Extract 3)—when building towards non‐surgical recommendations:^20^


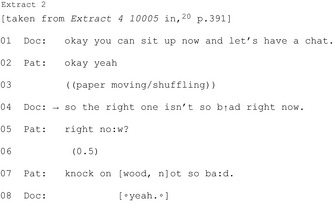



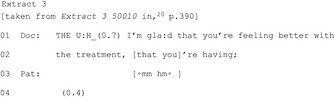


##### Logical inferences

Surgeons produced logical inferences which allow patients to infer non‐surgical recommendations:


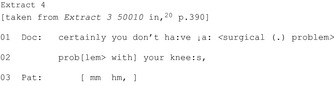


##### General/usual course descriptions

Describing the usual course for patients in general allows a patient to surmise the upcoming offer of non‐surgical treatment while also providing justification for it:


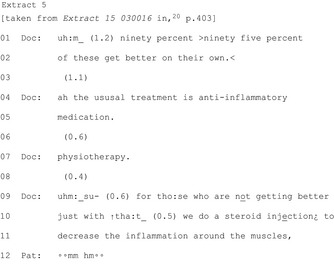


This “cluster of interactional devices are recurrently used in the lead‐up to recommendations not for surgery…[they] are designed to provide a persuasive argument for the upcoming recommendation” (p.1033).^27^ While most clear in surgical contexts, oncologists may also provide general rationale for adjuvant therapy (prior to a recommendation) by outlining how breast cancer has been treated historically compared to the current guidelines thereby strengthening the upcoming recommendation.^37^

##### Accounts

HCPs may pre‐emptively justify recommendations by providing accounts, such as oncologists accounting for adjuvant therapy in advance of recommendations for chemotherapy and/or radiotherapy to post‐operative breast cancer patients^37^ or consultants justifying moving from intensive to supportive/palliative care though accounts of professional consensus or those rooted in the idiom of the “best interest” of the patient (in this particular case, the baby).^38^ In a physiotherapy setting, therapists sometimes use accounts when the upcoming recommendation is counter to patient's expectation or report of current activity.^33^

In sum, these practices forecast and strengthen the recommendation/suggestion prior to its production. Used particularly when the recommendation is liable to resistance or counter to patients' expectations, they function to achieve patient agreement in a potentially challenging environment. Indeed, resistance was more likely when forecasting activities were absent.^20^

#### 3.1.4 Patient lobbying for specific treatment prior to commitment point

Prior to HCPs referring to specific courses of action or making commitment relevant, patients may reference a particular course of action to seek pre‐emptively to influence the treatment trajectory. Documented in five publications,^18^, ^25^, ^26^, ^41^, ^42^ this is the only patient/companion‐initiated broaching activity described in the included publications.

If a patient knows a diagnosis projects a treatment, challenging the diagnosis may be a way of lobbying for a desired treatment. In consultations with children with upper respiratory illnesses, parental resistance to a viral diagnosis may be a resource for resisting the projected non‐antibiotic treatment.^42^ This pre‐emptive subtle influence was also identified in two single‐case analyses. In a hospital outpatient clinic, by inquiring about the availability of a test and describing a previous positive experience in a similar situation with the doctor's predecessor, the “patient exerts subtle but persistent pressure for a diagnostic test” (p.451) before the doctor's recommendation.^25^

By lobbying, patients position themselves as having a role in determining the decision. Nevertheless, generally this pressure is applied subtly (attentive to being heard as possibly treading into HCPs' territory^41^) and designed not to oblige the HCP to offer or decline the lobbied for treatment/test.

### 3.2 Putting forward the course of action (the commitment point)

The next phase—although decision making may begin here if there are no broaching activities—is putting forward or ruling out possible paths of action. This is a *commitment point* as it obliges the patient to make or (implicitly or explicitly) avoid commitment. This activity is solely within the HCP's domain in the studies reviewed.

#### 3.2.1 Single option

The most common way HCPs reach a commitment point is by putting forward a single course of action (27 of 28 publications); these practices are imbued with varying levels of assumption that the patient should/will follow that course of action. A HCP may make an explicit recommendation (Extract 6) or even build in presumption of agreement (Extracts 7‐8).


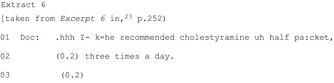



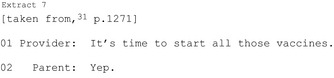



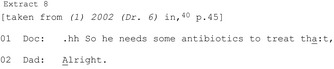


A HCP may produce a suggestion (eg “my suggestion would be…”)^45^ which conveys their stance but reduces their authority to require the patient take the particular action. They may be structured to indicate a shared decision (Extract 9) or entirely the patient's choice (Extract 10).


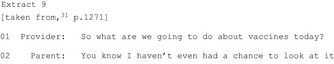



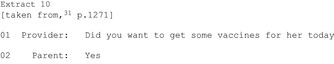


Putting forward a single path—however openly phrased—is likely to be heard as HCP‐endorsed (see^34^). Sometimes a HCP may be able to offer a single option only (likely to vary significantly depending on setting) and doing so does not preclude recognition of patient autonomy. These turns bring the interaction to a commitment point. Patient agreement here is sufficient to reach a decision. Although interactionally more difficult, patients may reject the course of action. This difficulty may be compounded by the format used (eg it is more difficult to reject announcements than suggestions).

#### 3.2.2 Ruling out a single option (primary treatment)

HCPs may specifically rule out a particular option (eight publications).^18^, ^20^, ^27^, ^28^, ^29^, ^39^, ^41^, ^42^ These appear less common than affirmative recommendations: in one study, initial recommendations against a treatment were found in 29 of 309 consultations (compared to 252 initial affirmative recommendations).^39^ Compared with affirmative recommendations, a narrower spectrum of formats are used to rule out including “I don't think we need to…”; “I don't recommend…”; and “you certainly do not need….”

An example from paediatric acute care shows a whole class of treatments being ruled out with “I don't think we need to put her on any medication.”^39^ More frequently (in all eight publications), the HCP rules out a particular treatment. From a primary care consultation, Extract 11 shows a doctor ruling out antibiotics:


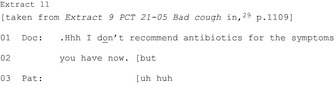


Seven of the eight publications show ruling out occurring in environments in which there is an orientation to a primary treatment (eg surgery, antibiotics). The ruled‐out treatment is treated as known to the patient, expectable and possibly preferred. Sometimes HCPs also offer an affirmative alternative (treated as less preferable). They usually occur after the rule out and as a result of the patient's response to it rather than designed that way from the outset.^39^

Often HCPs engage in activities for seeking agreement prior to ruling out the primary and/or offer an alternative to create an auspicious environment for patient agreement. The rule out might be produced as a temporary decision (Extract 11) which preserves the primary as a possible future option. Indeed, in an orthopaedic surgery consultation, the rule out is achieved by referencing surgery as on offer in the future (“delay your surgery”).^20^ Ruling out a course of action is less straightforward—both its design and reception—than affirmative recommendations.

#### 3.2.3 Multiple options

Less frequently—primary finding in five publications,^22^, ^36^, ^38^, ^44^, ^45^ subsidiary in one^30^—HCPs put forward multiple options from the outset (rather than offering options in response to withholding commitment to a single option). This practice (ostensibly) provides clear opportunity for patient participation. Usually, HCPs announce that multiple options are about to be listed, perhaps because otherwise the recipient might be primed to hear the first option as a single option. Multiple options tend to be presented with multiturn units detailing benefits, risks, effects or rationale of each of the options and with opportunities for patient responses. After the list, the HCP may elicit the patient's view (eg “what do you think”).^45^ The options may be fairly neutral or may display—strongly or weakly—a stance. In a neonatal ICU, some options were more persuasively presented than others.^38^ In mental health consultations, a psychiatrist flagged up three choices, discounted the first two before producing the third: the structure of offering multiple options can thus be a vehicle to recommend a single path.^36^ Another study showed that in six of 15 ICU cases, the presentation of options was “shaded”: not all options were present or physicians' preferences were strongly indicated.^30^

### 3.3 Committing or not

#### 3.3.1 Committing

The nature of the initiating action shapes what constitutes a relevant next action. A single option makes relevant commitment (or avoiding commitment), and lists make relevant a selection.^44^ When a single option is put forward (as is most common), patients/companions and HCPs jointly treat patient commitment as the necessary next action. Eleven publications^23^, ^24^, ^29^, ^30^, ^31^, ^37^, ^39^, ^40^, ^42^, ^43^, ^44^ attend to this explicitly, and in a further 11^18^, ^19^, ^20^, ^27^, ^28^, ^32^, ^33^, ^36^, ^38^, ^41^, ^45^ agreement as the necessary next step is presumed.

At the commitment point, all parties treat patient involvement as crucial, although this can involve very short utterances. Commitment involves accepting rather than merely acknowledging: “treatment recommendations are routinely accepted with objects such as period intoned ‘Okay.’ or ‘Alright.’; ‘Let's do that.’; ‘That's fine.’; and assessments such as ‘Good’” (p.46‐47).^40^ Patients may produce themselves as involved even if they say very little.

In addition, patients may *implicitly* commit by continuing to the next activity. In oncology consultations, a patient's implicit agreement is shown by moving to a question about treatment location, however, ‘very rarely…do these unfold with so little input from the patient’ (p.88).^37^ The severity of the condition and complexity of decision may have a strong bearing on this. In an ICU setting, consensus regarding decisions pertaining to removal of life‐sustaining equipment was a topic in its own right.^30^

Acceptance is only sufficient when a single path has been put forward. In cases of multiple options, the relevant next action is selection from the list. However, patients may challenge the option‐listing format by seeking a recommendation instead.^44^ In some cases, commitment to a course of action is not required in that interaction. Three oncology consultations were examined in which decisions regarding adjuvant therapy were left open.^37^ In these cases “visits are treated as opened‐ended sessions in which there is no expectation for an on‐the‐spot decision” (p.102).

#### 3.3.2 Withholding commitment

Patients/companions may withhold commitment through silence or weak commitment, regarded as “tacit resistance.”^31^ This stalls progress and implies a problem but does not specify the nature of the problem. It is not indicative of definite or enduring resistance: there may be obstacles to overcome before the patient commits. In primary care consultations, patients withheld commitment until they were clear what was being proposed; certain the recommendation was complete; or sure what the recommendation meant.^29^

Nine^19^, ^23^, ^29^, ^31^, ^37^, ^39^, ^40^, ^42^, ^43^ of the 28 publications explicitly discuss how patients/companions withhold commitment, often referring to this as “passive resistance,” with a further 12 publications^18^, ^20^, ^27^, ^28^, ^30^, ^32^, ^33^, ^36^, ^38^, ^41^, ^44^, ^45^ making implicit reference. Withholding commitment obliges HCPs to stay within the decision making phase however, if commitment is still not achieved, patients may move to “active resistance.”

#### 3.3.3 Active resistance

After a commitment point has been reached, the patient/companion may question or challenge the proposed course of action. In the publications reviewed, this “active resistance” occurs as an escalation from initially withholding commitment. Two main practices for actively resisting were identified.

##### Questions/concerns

First, patients may raise questions or concerns about the medical problem or the proposed treatment/plan (six publications).^19^, ^29^, ^31^, ^37^, ^38^, ^40^ Extract 12 shows a patient questioning the diagnosis (lines 19‐21, 27‐29 and 31‐32) and raising a previously unarticulated concern (lines 45‐46).^29^ These instances of active resistance often indicate the nature of the barrier to commitment.


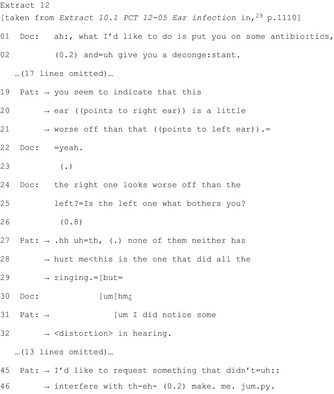


By questioning, patients assert themselves as involved participants by providing opportunities for both parties to negotiate what constitutes acceptable treatment/plans. The extent to which questioning is heard as challenging may vary depending on the way the commitment point was reached. In a neonatal ICU, questions after recommendations were heard as more challenging than those after multiple options were given.^38^

##### Advocating for some alternative after reaching a commitment point

Identified in five publications,^23^, ^31^, ^36^, ^39^, ^42^ the second—stronger—form of active resistance is advocating explicitly for some alternative. Examples of these include a parent requesting just two of the three scheduled vaccinations;^31^ a patient in a mental health setting advocating for medication dosage change immediately rather than in the future as the doctor has suggested;^36^ and in a GP consultation, a patient suggesting a lower dose than the doctor proposed.^23^

### 3.4 HCPs' responses to patients' resistance or withholding of commitment

In instances of resistance or withholding commitment, HCPs engaged in three possible actions: pursuing without changing course; modification of the proposal; and/or leaving open the decision for future revisiting.

#### 3.4.1 Pursue agreement without changing course

Eight publications^18^, ^20^, ^29^, ^31^, ^32^, ^36^, ^37^, ^40^ document pursuit of commitment after resistance. In five of these, pursuit involves treating patients' problems as obstacles to overcome before agreement is achieved. Patient participation is evident despite the unchanged treatment trajectory as patients “postpone acceptance until their treatment preferences and concerns are satisfied” (p.1110).^29^

The remaining three publications show HCPs pursuing commitment after resistance without engaging with the patient's/parent's problem as in Extract 13 showing a paediatric vaccination consultation:


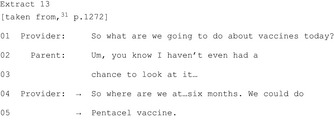


In another of these three publications, a doctor pressurizes a patient to commit to a medication change in a mental health consultation.^36^ The persuasion is strong, the patient orients to it as pressure, and their eventual agreement is grudging. Yet the analysis shows that the patient engages in activities (eg retrospectively orienting to doctor's recommendation as advice, reluctant agreement) which convey that a shared decision is taking place.

#### 3.4.2 Modify the potential course of action (pursuing agreement by changing course)

HCPs may modify the course of action (five publications).^23^, ^29^, ^36^, ^40^, ^41^ Taken from an oncology consultation, Extract 14 demonstrates a dosage recommendation modified to a recommendation to “work up to” that dosage and furthermore only “if you can”:


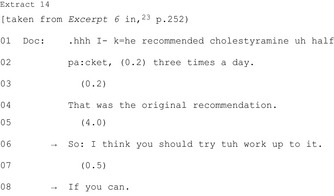


These strategies do not operate in isolation, and HCPs may begin with pursuit then shift to modification. In a mental health consultation, a psychologist offers an alternative medication after many minutes of attempting to persuade the patient to commit to the original recommendation.^36^

These modifications usually involve giving the option of declining the treatment or taking a lower dose, or providing an alternative but equivalent treatment. However, there was one phenomenon, reported in two publications from the same programme of research, in which children diagnosed with viral respiratory illnesses therefore recommended non‐antibiotic treatments were subsequently prescribed antibiotics as a result of parental resistance.^40^, ^41^ Clearly, in these instances, parents are active participants. However, if this results in action counter to the diagnostic trajectory, then this goes beyond *shared* decision making.

#### 3.4.3 Leave the decision open

Documented in four publications, HCPs may attend to patients'/companions' resistance by leaving “open” the decision, either by deferring it until another time^23^, ^30^ or by offering to review/revise at a later date.^31^, ^40^ In an oncology consultation, resistance to gall bladder removal resulted in a recommendation to “think about” surgery in the future,^23^ and in an ICU, lack of consensus about withdrawing a relative's ventilator resulted in deferral until agreement can be reached.^30^ Two publications show doctors offering the option of revisiting the decision if it turns out to be unsuitable.^31^, ^40^ The offer of revisiting the decision is in response to resistance, that is, these are distinct from interactions that are designed from the outset as not requiring commitment during the encounter or framed from the outset as temporary.

## 4 DISCUSSION

This systematic review included 28 publications reporting CA studies of decision making in health‐care encounters. The review mapped 13 communication practices across four decision‐making elements. These four are not arbitrary: using the HCP's turn in which commitment to a course of action becomes relevant as a pivot around which the other activities are arranged is an expedient organizing feature—for HCPs and patients—that makes clear what different activities are relevant at various points. The review offers an overall framework within which to situate other decision‐making activities. We have shown that communication practices may be subtle and even if they ostensibly look similar may not function in the same ways. We note that patients may enact their agency throughout the encounter, but sequences outside the decision making phase are beyond the review's scope.

There is wide support for SDM,^2^, ^3^ and the findings included in this review concur with this. We have explicated practices that contribute to SDM occurring in a range of settings. The review shows information exchange, although an important aspect of SDM,^2^ is only useful if information is recognized and incorporated into the decision‐making process. This is a time‐consuming activity but seeking to understand the constraints and complexity of this activity “seems a logical step in developing rigorous and comprehensive knowledge, appropriate practice and useful professional guidance about this area of clinical communication” (p.679).^32^

There has been little focus on multiple options compared to single recommendations. Option listing conveys a different relationshp between HCP and patient “because the [HCP] is claiming—when option‐listing—only to know what options are available, not which one the patient should take” (p.13).^44^ By offering multiple options, HCPs surrender some of their authority. However, we have explained that this practice does not guarantee patient‐led choice.^44^ Existing research suggests that SDM is not always happening, despite HCPs' claims,^7^, ^46^ and offering multiple options in such a way as to actually pursue a single course is an example.

For some decisions, there is a single obvious course of action but this is not the case for most health‐care decisions^47^ and these vary according to settings. For example, counterproposals were found in GP consultations, but not in oncology clinics where recommendations tend to be protocol driven so modification is less likely.^23^ Scope for a patient to influence the treatment trajectory is limited when an option such as surgery^20^ or antibiotics^39^ is ruled out. But even where the patient's influence over the decision is limited, HCPs can work to involve patients through seeking to bring them to a point of shared rationale. The operation of this practice may be setting specific. In paediatric consultations, resistance may be less likely if rule outs are followed by affirmative recommendations for alternative treatments.^39^ Whereas, in surgical environments, ruling out surgery may be more acceptable than affirmative non‐surgical alternatives as it shows patients' problems have been taken seriously and considered in relation to surgery.^20^

### 4.1 Limitations

The categories identified are broad because the 28 publications span a wide variety of conditions: acute to chronic; minor to life‐threatening; those with multiple treatment paths to those for which protocols or urgency dictate one path. The evidence—while rich in detail—is concentrated in specific areas (eg acute care, particularly antibiotic prescription; orthopaedic surgery; oncology). Practices may operate differently depending on the setting (eg ruling out) which can be problematic for transposing findings to other settings. Therefore, we offer the practices presented here as a mapping rather than a definitive structure. However, despite differences, common activities exist.

We attempted to weave appraisal findings into our syntheses but it is difficult to assign relative weight to CA studies' findings due to their qualitative nature and associated small data sets.

For practical reasons, we included English language publications using English language data only. This is disappointing as several excellent studies using non‐English language data would contribute to the findings, for example Norwegian work exploring differences across different health‐care settings^48^ or analysis of Swiss physiotherapy data which contributes to understanding goal elicitation.^49^ Similarly, we did not include grey literature.

### 4.2 Applications for practitioners

By lobbying for specific treatments/plans, patients produce themselves as having a role in determining the decision. The subtlety of this lobbying orients to the delicacy of potentially stepping into the professional's domain. Patients' requests therefore may not look like typical requests, and practitioners can be responsive to this without having to grant the request while also providing reassurance.^26^ Patients' resistance often provides opportunities for HCPs to address specific problems, thereby treating them as involved participants. Patients are skilled at doing this in ways that avoid confrontation, and it would be beneficial to HCPs to be able to recognize these.^19^

Eliciting patient perspectives and ensuring that information is genuinely taken into consideration generally result in patients experiencing themselves as involved. Practices such as relatively lengthy HCP turns, HCP talk, focusing on positives, intimating/accounting for upcoming recommendations and descriptions of general cases work to seek patient agreement prior to HCPs' recommendations. These practices are typically used when recommendations are counter to patient expectations/preferences, and work to increase the likelihood of patient commitment. For HCPs, this strategy is particularly useful when there is only one option and therefore little scope for alternatives because it treats patients' full commitment as important. However, where there are multiple viable options, practices that work to encourage patient agreement with a particular option could curtail the patient's opportunity for choice and participation.

Ways of putting forward a single course of action lie on range from asserting/informing to offering. Whether asserted or offered though, when HCPs present a single option, this option is likely to be heard as HCP‐endorsed. Giving multiple options may increase patients' perception of participation, but, if options are limited to exclude viable possibilities or options are strongly weighted (eg^36^, ^45^), this practice may operate as a vehicle for recommending.

Recognition that patient resistance is a resource for participation means that using the interactional slot after resistance to invite patients to collaboratively construct an acceptable decision is “a candidate best practice” (p.1111).^29^ Exploring patients' reasons for resistance—even when protocol means there is no alternative—validates patients' participation. Even where the patient eventually agrees to the original recommendation, where reasons are explored, they will have still participated in the decision making process. Pursuing agreement without engaging with patients' reasoning for withholding is less encouraging of patients' participation and may be treated as coercive. Where the option for modifying recommendations is possible, this allows for greater patient participation in terms of influencing the final decision. However, as patients/companions become increasingly proactive in their health‐care, HCPs balance the encouragement of participation with the importance of need to not being pressured to give inappropriate treatment.^41^

### 4.3 Future research

Finally, we discuss three opportunities for future research. First, HCPs give treatment and decision relevant information at various points (eg prior to recommending, during offering single/multiple options, after patients withhold commitment) but this has received limited attention as a phenomenon in its own right. This is particularly important as information sharing is central to patient participation. Second, existing studies (particularly those with extensive data sets) have been concentrated in a few specific areas, for example primary care. Given that setting and condition can shape the operation of these practices, it would be valuable to explore a range of secondary care settings and also settings in which successful outcome is arguably more subjective—such as maternity care, palliative medicine or plastic surgery. Third, the actions that we have outlined here may be achieved by a range of practices that has not yet been fully documented.

## 5 CONCLUSION

Decision making encompasses more than the turn in which a course of action is put forward and patient's immediate response to it. Understanding of decision making can usefully be arranged around the commitment point because everything done after this point is it commitment relevant. Where there are multiple viable options, there are a number of ways of encouraging patient participation in reaching a shared decision. Putting forward only a single option provides for patient input because once that option is on the table, HCPs do not move on to other phases of the consultation until the patient's has made some verbal commitment. Even when it is not possible for patients to influence the treatment trajectory, the spirit of SDM can be invoked by incorporating practices that encourage patient participation in particular by deploying practices that aim to equalise the patient's understanding of the rationale of the trajectory with the HCP's understanding.

## CONFLICT OF INTEREST

There are no conflict of interests to declare.
